# The Use of Polyimide as a Bonding Material to Improve the Mechanical Stability, Magnetic and Acoustic Properties of the Transformer Core Based on Amorphous Steel

**DOI:** 10.3390/polym16131840

**Published:** 2024-06-28

**Authors:** Jolanta Nieroda, Grzegorz Kmita, Michal Kozupa, Szymon Piela, Andrzej Rybak

**Affiliations:** 1 ABB Corporate Technology Center, Starowislna 13A, 31-038 Krakow, Poland; jolanta.nieroda1@pl.abb.com; 2 Hitachi Energy Research, Pawia 7, 31-154 Krakow, Poland; grzegorz.kmita@hitachienergy.com (G.K.); michal.kozupa@hitachienergy.com (M.K.); szymon.piela@hitachienergy.com (S.P.)

**Keywords:** polymer binder, polyimide layer, metallic glass, surface characterization, electrical devices, electric losses, noise analysis

## Abstract

The constantly evolving electrification also entails an increase in requirements for the effective and efficient distribution of electricity with the lowest possible power losses. Such needs can be met by highly effective electrical devices, and one of them is a transformer whose main component is a magnetic core. Currently, one of the soft magnetic materials used alternatively for the production of transformer cores are amorphous metal strips with competitive losses. However, to successfully use these materials, a key problem must be solved: limited mechanical stability. The presented article describes the development and application of a polyimide-based binder for efficient bonding of an amorphous metal ribbon. The layered binder was characterized using confocal microscopy, scanning electron microscopy and Raman spectroscopy, and its anticorrosion and mechanical properties were examined. As a final step, a prototype of a toroidal magnetic core bonded with the binder was manufactured and subjected to the evaluation of no-load loss and the analysis of the emitted noise. It was confirmed that the proposed polyimide binder tremendously improved the mechanical stability while reducing core losses and audible noise.

## 1. Introduction

The electrical energy consumption is growing day by day due to the higher amount of electricity dependent equipment used by the human population [1,2]. Such a high energy consumption in almost every corner of the world forces the production of highly efficient devices that will be able to transfer energy over long distances and adjust the voltage to people’s needs. Such an apparatus is a transformer, the main part of which is a magnetic core made of ferromagnetic material [3,4]. In the beginning transformer cores were made of shaped iron. Then, in order to improve magnetic properties, this material was replaced with isotropic hot-rolled steel, subsequently an anisotropic grain-oriented steel, a high-permeability steel, a domain-refined steel, a microcrystalline steel, through a nanocrystalline steel, and recently the amorphous steel called also metallic glass has come into use [5,6].

Amorphous metal is a material with lack of long range order of atoms and it is received from a melted alloy solidified by rapid cooling (10^6^ K/s) [7,8]. A final product is a thin, for example 25 μm, ribbon which is characterized with an excellent magnetic properties, like easy magnetization and high magnetic permeability [9,10]. A consequence of easy magnetization are low hysteresis losses which are the major problem observed in the transformers cores. The second type of core losses are the eddy current losses which are also reduced in the amorphous steel core as this core material exhibits high resistivity [11,12]. As a result, the total core losses are as much as 70% lower than in the previously used core material, namely a regular grain-oriented (RGO) steel [13].

Despite great advantages of using the amorphous steel for the core in transformers, this material exhibits few disadvantages. The result of the rapid cooling is quenching stresses that can be reduced by the annealing process [14,15]. Moreover, during annealing an external magnetic field is applied and magnetization axis is introduced in the material, therefore it is an essential step. The negative consequence of annealing process is that the amorphous steel becomes brittle and is problematic in the further processing. Another disadvantage of the transformer core made of the amorphous steel is from 3 to 5 dB higher the sound level in relation to the transformer core made of RGO steel [13]. This sound is a result of magnetostriction effect, during which the magnetic material changes dimensions under the influence of a magnetic field [13]. In order to reduce the presented disadvantages, a solidifying agent in form of binder can be applied between the laminates [16].

The binding material has to be adequately selected. At first, binder should be safe for the steel substrate, namely it cannot cause or even prevent its degradation such as corrosion. Secondly, it should possess possibility to wet the surface and build a durable bond with the substrate. Finally, the chosen material should create a good quality layer without cracks and with appropriate parameters.

Due to its application in the magnetic core, the binding layer should be a dielectric material and do not cause deterioration of magnetic properties. Moreover, synthesis and/or processing of such binder must be completed below the Curie temperature of the magnetic ribbon, which disqualifies many attractive materials like ceramics or glasses. At the end, it should withstand an elevated temperature that is applied during the orientation process of magnetic domains.

Therefore, the research presented here proposes the evaluation of the binder based on a polyimide polymer layer. Polyimide is characterized by chemical resistance, thermal and dimensional stability [17], it possesses high mechanical properties [18], flame resistance and good flexibility [19]. The combination of such properties leads to industrial applications such as aerospace, automotive or microelectronic [20], in the form of adhesives, sealants or wire coatings.

In the literature one can find several works containing recipes for obtaining polyimide (PI), and it is usually produced by a two-step process. At the beginning, a polyamic acid precursor is synthesized in aprotic polar solvent. In the second stage, a polyamide acid is cyclized through the chemical or thermal imidization [20]. Unfortunately, the popular aprotic polar solvents for polyimide synthesis are N-methyl-2-pyrrolidone, dimethylacetamide and dimethylformamide and all of them are classified as hazardous for the respiratory system [21,22,23]. On the other hand, it is possible to find a promising work where polyimide powder was dissolved in dimethyl sulfoxide (DMSO) [24]. This solvent is not hazardous, and additionally it is environmentally friendly and it is also popular in the food industry.

Only a few literature positions can be found regarding binding of the amorphous metal cores, and they are mainly patent applications. The topics related to insulating properties of binder and its solidification and strengthening influence on the magnetic core for its easier transportation are topics known for many years. It can be found the works in which the magnetic core was solidified by edge bonding with resin [25], but also with ceramic coating [26]. The edge solidification solves the transport problem of brittle amorphous core after the annealing process. Next two patents present the ways of resin application directly on the amorphous metal, in order to cover substrate with insulating material [27,28]. The most interesting positions are where binder application was used in order to reduce noise related to the working core. First of them presents application of high strength, dielectric tape on columns of the core [29], while the second describes usage of organometallic binder based on alkoxysilanes [16]. In both cases, the binders fulfill their role and reduce audible noises of prepared magnetic core from the amorphous metal.

The presented article describes the application of polyimide solution on the amorphous metal ribbon, in order to receive the mechanically stable magnetic core with the reduced noise and decreased magnetic losses. The deposited layer was characterized by means of Confocal Microscopy (CM), Scanning Electron Microscopy (SEM) and Raman spectroscopy. Additionally, the adhesion properties of the received binder was tested, and also anticorrosive character and dielectric properties were evaluated. Finally, a prototype of a small, toroidal magnetic core was prepared and examined for noise and magnetic losses.

## 2. Materials and Methods

### 2.1. Amorphous Metal Ribbon

The main material used in the study was 2605HB1M amorphous metal ribbon from Metglas, Inc. (Conway, AR, USA), characterized by two different sides, namely glossy and matte, as shown in Figure S1. The 25 ± 4 μm thick ribbon is an iron-based alloy consisting of (by weight) 1–10% boron, 85–95% iron and 1–10% silicon [13], and is produced by rapid cooling (10^6^ °C/s) of molten alloy, which results in an amorphous nature and lack of long-range order [30].

### 2.2. Preparation of Polyimide Layers

Solution of polyimide (PI) was prepared by dissolution of P84 polyimide powder (100%, HP Polymer GmbH, Ontario, Canada) in dimethyl sulfoxide (DMSO, 100%, POCH, Gliwice, Poland). The amorphous ribbon was degreased with acetone prior PI layer deposition. The most optimal thickness of coupling layer is 4 µm between two pieces of amorphous ribbon, therefore single layer on glossy or mat side should be equal to 2 µm. In order to receive such thickness, a 10% by weight solution was prepared and deposited with special steel bar that left 20 µm layer after deposition. DMSO possess high boiling temperature, namely 190 °C, and relatively low self-ignition temperature at 215 °C, therefore drying in a furnace is dangerous and cannot be used. In order to dry the deposited layers a heat gun was used. The pre-dried samples do not possess coupling properties, therefore they need to be heated up in order to make polymer soft. As the transformer core need to be annealed at temperature 320 °C for 1h in magnetic field, such temperature and time were used as annealing process parameters for deposited layers. The deposited, pre-dried and annealed samples are presented in the Figure S2.

### 2.3. Confocal Microscopy

The CM examination was performed with the LEXT OLS4000 confocal microscope (Olympus, Tokyo, Japan), equipped with 405 nm laser. The biggest possible testing area (6.64 mm^2^) was selected, and with used 5× objective lens, the final image had 108× magnification. Investigation of the surface roughness was performed with the mean deviation of the assessed profile parameter (R_a_) according to the ISO 21920-2 standard [31]. The R_a_ parameter was calculated as an average of five perpendicular and five parallel surface roughness profiles. The example of lines distribution on the tested area is presented in the Figure S3.

### 2.4. Scanning Electron Microscopy

SEM investigation was performed with the NOVA NANO SEM 200 scanning electron microscope (FEI Europe B.V., Eindhoven, The Netherlands) coupled with the EDX detector (EDAX, Mahwah, NJ, USA). Because the tested ribbon is a conductive material, therefore samples were analyzed without additional pretreatment, and with 15 kV of accelerating voltage.

### 2.5. Raman Spectroscopy

Raman spectroscopy measurements were performed by means of ex-situ method with LabRam HR (Horiba, Kyoto, Japan) spectrometer equipped with 633 nm laser. In all cases 4000–50 cm^−1^ range, 1800 grooves/mm and two accumulations were applied, while acquisition time was adjusted between 10–30 s.

### 2.6. Mechanical Examination

Binding strength of PI layer was tested with tensile measurements of coupled pieces of amorphous metal ribbon. Samples were cut to the size 7 × 2 cm, and were covered with binder, then pre-dried with the heat gun and connected together with the 2 × 2 cm of a bound area (see Figure S4). Next, they were placed between two flat steel elements and cured for 1 h at 320 °C. Samples were examined at room temperature by testing machine Instron 3367 (Instron, Opole, Poland), with applied 1 mm/min tensile speed. Binding strength was calculated from noted maximum load before the failure and referred to the bonded area.

### 2.7. Corrosion Resistance

The corrosion resistance was evaluated by means of simultaneous thermal and humidity aging of samples in the climatic chamber MKFT-115 (Binder, Tuttlingen, Germany). Before insertion into the chamber, the amorphous steel samples with size 7 × 2 cm were covered in half with a binder, and then hung vertically on a stand. The samples were subjected to the temperature of 120 °C and humidity 45% RH, what resulted in an environment fully saturated with water vapor. The corrosion resistance test was lasting for 30 days, and samples were analyzed after 15 and 30 days.

### 2.8. Dielectric Measurements

Investigation of the dielectric parameters was done with use of sample arrangement with the following condenser-like structure: amorphous steel tape/binder layer/silver electrode, as shown in the Figure S5.

A Broadband Dielectric Spectrometer with Alpha-A High Performance Frequency Analyzer (Novocontrol Technologies, Montabaur, Germany) was used for evaluation of the dielectric parameters. The samples were sandwiched between two copper electrodes and placed inside a temperature controlled sample cell, as shown in the Figure S6.

The complex permittivity:

(1) $$\varepsilon^{*}\left(f\right)=\varepsilon^{\prime }\left(f\right)+\varepsilon^{\prime \prime }(f)$$

where:

ε*(f)—complex permittivity [F/m],
*ε′(f)*—real part of complex permittivity [F/m],
*ε″(f)*—imaginary part of complex permittivity [F/m],

was determined in the frequency (f) range from 10^−2^ to 10^7^ Hz and in the temperature range from 25 to 100 °C with 25 °C step. The alternating-current voltage applied to the capacitor was equal to 3 V.

### 2.9. Preparation of Toroidal Magnetic Core

**Figure 1:**
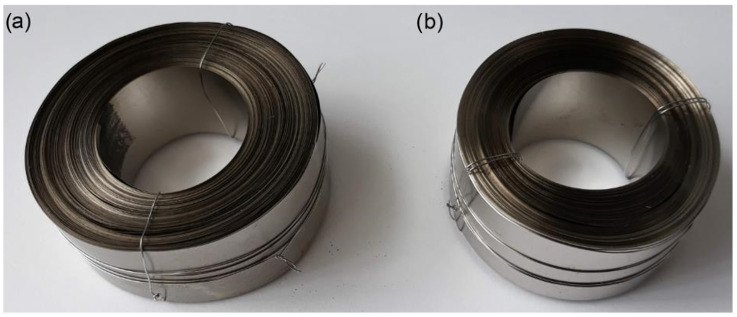
Small toroidal magnetic core: (**a**) bonded with PI, (**b**) reference.

In the second part of examination a magnetic core consisting of 5300 mm amorphous ribbon was prepared. At the beginning ribbon was pretreated with paper soaked with acetone. In the next step, PI solution was deposited on amorphous steel with steel rod. After that, ribbon was pre-dried with heat gun, and after rolling it was annealed at 320 °C for 1h. The final toroidal cores are presented in the Figure 1. The cores prepared in this way were examined for their magnetic losses and also noise analysis was performed.

### 2.10. Magnetic Measurements

The AMH-1M-S Permeameter (Laboratorio Elettrofisico, Nerviano, Italy) was used for determination of hysteresis loop, remanence *B_r_*, coercivity *H_c_*, saturation values *H_s_*, *B_s_*, and magnetic losses. It is a DC and AC automatic measuring system used for characterization of the soft magnetic metal rings and strips at a high resolution. The toroidal core has to be prepared by winding of a primary set of turns *N_H_* (Drive) around the sample for excitation and a secondary set of turns *N_B_* (Sense) to detect the magnetic flux (see Figure S7). The *H*(*t*) field is determined measuring the current *I*(*t*) in the primary winding:

(2) $$H(t)=\frac{N_{H}\cdot I(t)}{l_{m}}$$

where: *H*(*t*)—magnetizing force [A/m], *N_H_*—number of turns, *I*(*t*)—current [A], *ℓ_m_*—mean magnetic path length [m], if external diameter of core *D_e_* does not exceed internal diameter of core *D_i_* more than 10% the value of *ℓ_m_* can be approximated with the mean circumference:

(3) $$l_{m}=\pi \frac{\left(D_{e}+D_{i}\right)}{2}$$

and if *D_e_* >> *D_i_* then *ℓ_m_* can be calculated as:

(4) $$l_{m}=\pi \frac{D_{e}-{\;D}_{i}}{\ln\left(D_{e}/D_{i}\right)}$$

The current is measured by determining the voltage across a low-inductance resistance *R* (Shunt)—see scheme in the Figure S7. The secondary winding produces the induced voltage *V*(*t*) from which the magnetic flux *Φ* is obtained:

(5) Φ=−∫V(t)dt

The magnetic induction *B* is then obtained by the relationship:

(6) $$B=\Phi /N_{B}\cdot A$$

where *A* is a cross-section of the magnetic core.

### 2.11. Noise Test Setup

The measured object was placed on the reflecting floor with vibroisolating rubber-cork mat to prevent any parasitic noise happening in the floor contact. The microphone was placed 30 cm above the measured object. A sound and vibration analyzer SVAN 958 (Svantek, Warsaw, Poland) was used to measure a Sound Pressure Level (SPL) with frequency analysis and time domain record. Before each measurement, the background noise was recorded. The AC Power supply PCR 1000 LA (Kikusui, Yokohama, Japan) was used in order to energize the core during the acoustic measurements, whereas MSO 4054 (Tektronix, Beaverton, OR, USA) oscilloscope was employed to analyze the quality of power delivered to the core. See images of acoustic test assembly shown in Figure S8.

## 3. Results and Discussion

The CM, SEM and Raman spectroscopy was used for investigation of three types of samples, namely: reference amorphous ribbon (marked as: Reference_g, Reference_m), samples with applied polyimide binder after pre-drying with the heat gun (marked as: PI_dr_g, PI_dr_m), and samples with PI layer after annealing at 320 °C (marked as: PI_an_g, PI_an_m). The abbreviation “g” means results for glossy side, whereas “m” refers to matt side.

### 3.1. Confocal Microscopy

**Figure 2:**
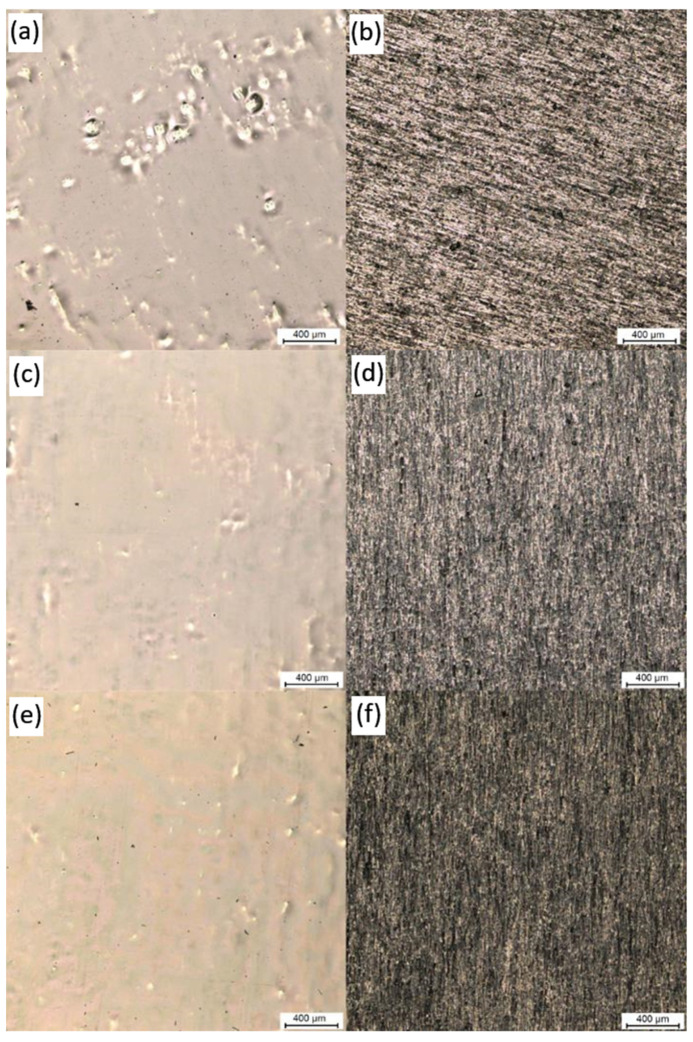
Confocal microscopy of samples (**a**) Reference_g, (**b**) Reference_m, (**c**) PI_dr_g, (**d**) PI_dr_m, (**e**) PI_an_g, (**f**) PI_an_m.

The overall surface state of polyimide layers was received after characterization with confocal microscopy and results are presented in the Figure 2.

It can be seen that received layers are continuous and crack free, both pre dried (Figure 2c,d), and even an elevated temperature does not destroy the film (Figure 2e,f). Confocal measurements were used also for calculation of surface roughness (R_a_) and results are presented in the Table 1.

It can be seen that in both cases, glossy and mat, deposition of PI films increases the surface roughness (PI_dr_g, PI_dr_m). After annealing film on glossy side becomes more uniform as the surface roughness is decreased (PI_an_g). Moreover standard deviation was lowered twice what confirms that annealing process leads to more uniform PI layer. On the other hand, no changes in surface roughness was observed on the mat side after annealing process (PI_an_m).

### 3.2. Scanning Electron Microscopy

**Figure 3:**
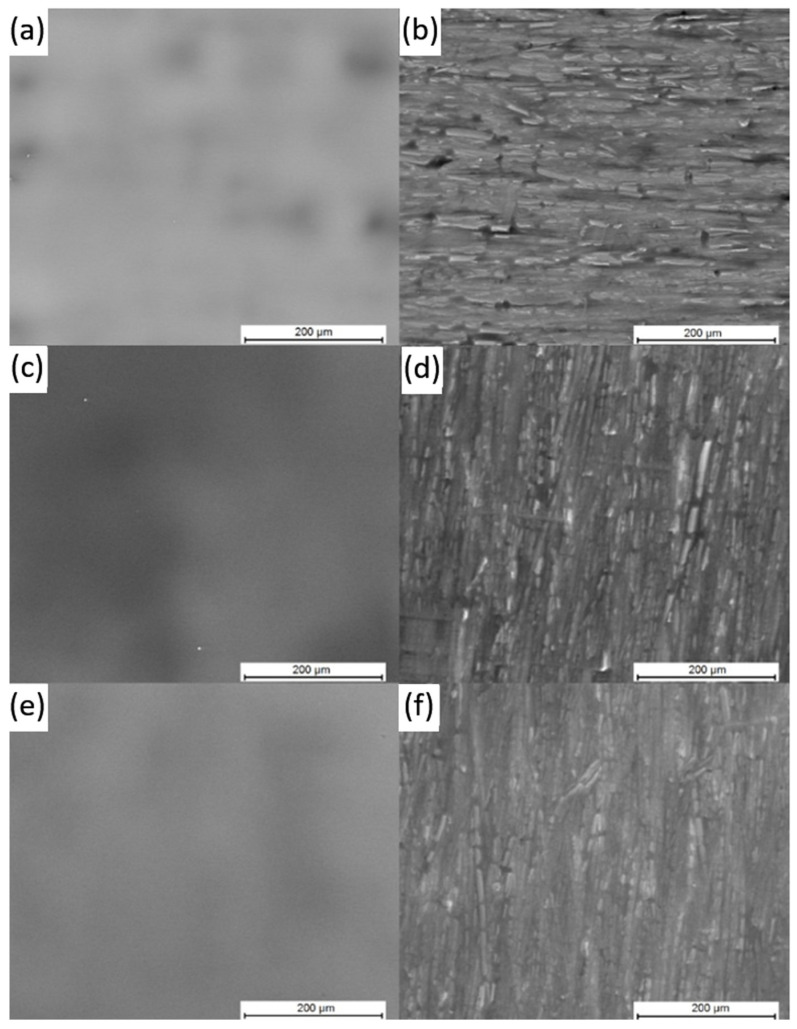
SEM examination of samples: (**a**) Reference_g, (**b**) Reference_m, (**c**) PI_dr_g, (**d**) PI_dr_m, (**e**) PI_an_g, (**f**) PI_an_m.

To be more precise in surface state examination, the SEM investigation was performed and the obtained results are presented in the Figure 3.

It can be observed that in both cases, for the pre-dried samples (Figure 3c,d) and annealed samples (Figure 3e,f), the obtained PI layers are continuous and crack free what confirms that the used drying procedure is adequate in order to manufacture PI layers, and the stress during formation is low enough to remain the deposited layers continuous and without defects. The SEM examinations were enhanced by the EDX measurements, and the obtained chemical composition is presented in the Table 2.

It can be concluded that polyimide binder layer is thick what can be deduced from a low amount of Fe detected during examinations, as presence of Fe is a response form amorphous steel surface. The Si presence, similar to the Fe, is only a response from the substrate. The highest noted amount is related to the carbon as it is dominating in the PI polymeric structure. In case of pre-dried samples (PI_dr_g, PI_dr_m), also S element was detected. Presence of the sulfur confirms that DMSO solvent was not fully evaporated during pre-drying with the heat gun. Nevertheless, its amount is very low and thus it should not be dangerous during annealing process in terms of low self-ignition temperature of DMSO. Therefore, the EDX examination confirms that the applied drying and annealing procedures for the deposited PI layer are correctly optimized.

### 3.3. Raman Spectroscopy

**Figure 4:**
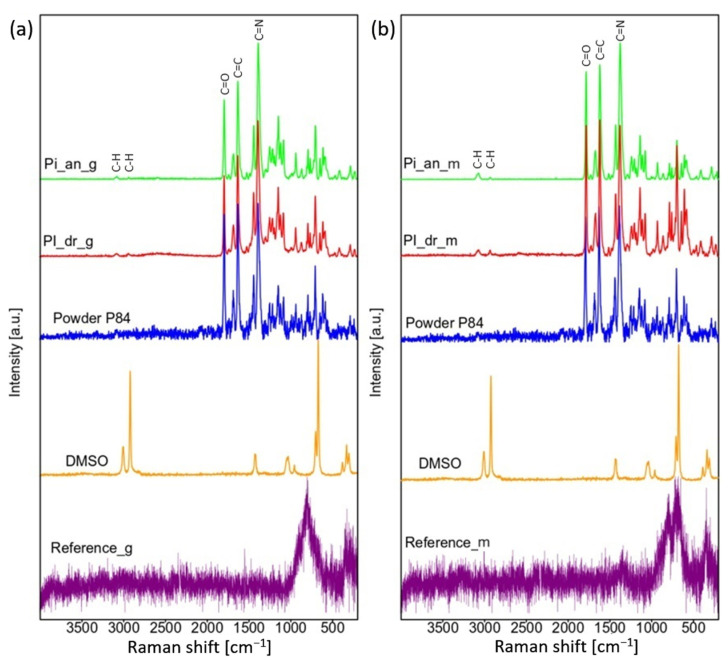
Raman spectroscopy of: (**a**) glossy, (**b**) mat samples covered with PI binder.

The results of Raman spectroscopy examination are presented in the Figure 4. It can be observed that in both cases, glossy and mat, samples covered with PI have a layer thick enough as there is no visible signal from the steel substrate. Despite the fact that in the SEM investigation there was no response from DMSO on annealed samples, it can be observed in Raman spectroscopy the presence of C-H bands at 3076 cm^−1^ and 2930 cm^−1^ for both, dried (PI_dr_g, PI_dr_m) and annealed (PI_an_g, PI_an_m) samples [32]. Nevertheless, the intensity of these bands is very low, what indicates that solvent residues are negligible. Rest of the bands noticed for dried and annealed layers can be attributed to the polyimide as presented spectra are very similar to spectrum of PI powder. Bands at 1783 cm^−1^, 1619 cm^−1^ and 1376 cm^−1^ indicate the C=O, C=C and C=N bonds respectively [33].

### 3.4. Mechanical Examination

**Figure 5:**
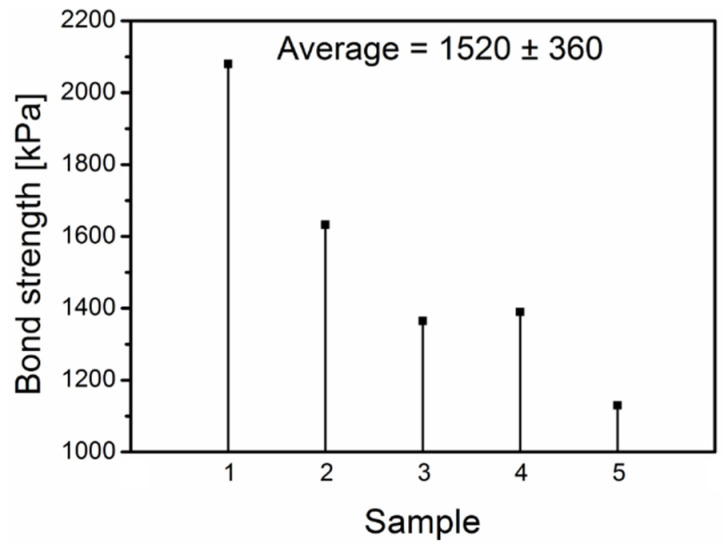
Tensile examination of metal ribbons bonded with PI layer.

The coupling strength of polyimide based binder was evaluated with tensile strength examination and the obtained results are presented in the Figure 5.

**Figure 6:**
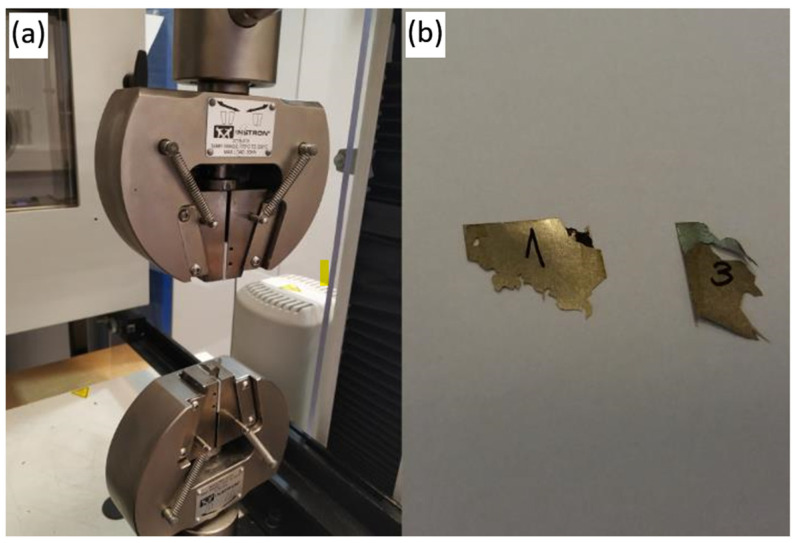
Sample bonded with PI after test: (**a**) remained part in tensile machine, (**b**) small pieces left due to cracking during test.

It can be seen that strength of bonding with PI is high, however also standard deviation is high. Such effect can be attributed to the non-uniform deposition of polyimide solution. Therefore, one can expect that in case of optimized mass production with the applied automated mechanical layer deposition, the standard deviation will be reduced. High strength of bonding can be confirmed also by the fact that samples were not simply decoupled after tensile examination, but they were entirely destroyed and split into many pieces. It proves that coupling area with applied PI binder was not the weakest part but rather the amorphous steel itself. State of the sample after tensile examination is presented in the Figure 6. It should be emphasized that in our previous studies on a silane-based binder, which has a lower bonding efficiency [34,35], it was observed that the metal samples debonded without destroying the sheet. Based on this, it can be concluded that the use of PI gives us a very efficient binder for improving mechanical stability of amorphous steel core.

### 3.5. Corrosion Resistance

**Figure 7:**
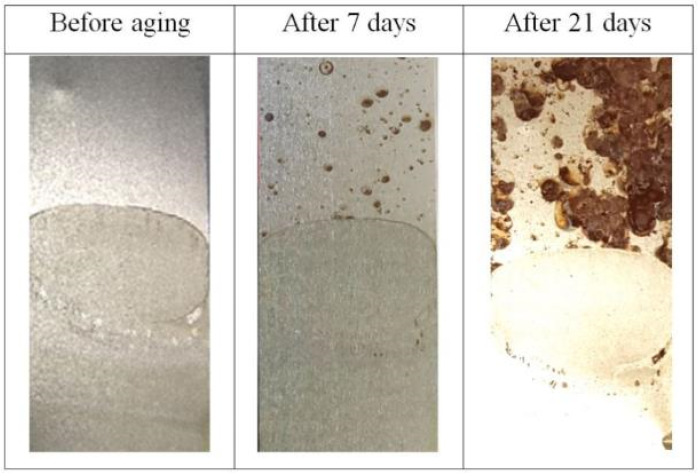
Images of samples treated partially with PI layer before and after aging in climatic chamber. Bottom half was covered with polyimide binder.

Samples coated with polyimide layer, pre-dried and annealed were inserted into climatic chamber in order to evaluate behavior in corrosive atmosphere. Results are presented in the Figure 7.

It is clearly seen that after aging for one week the corrosion was initiated in numerous points in the zone of raw steel tape (brown points), whereas part covered with the binder is free of corrosion. Very well developed corroded state can be seen on the surface of the sample which was aged for period of three weeks. The strong difference is visible between coated surface with no changes and raw surface with clearly seen the highly corroded areas. The observed effect shows that the polyimide based binder leads to enhancement of the corrosion resistance of the amorphous steel tape. Such effect can be used in order to improve the steel tape stability, e.g., prolonged shelf lifetime. Additionally, the electrical devices fabricated on the basis of the PI bonded amorphous steel tape will have increased corrosion resistance what is very beneficial from application point of view.

### 3.6. Dielectric Measurements

**Figure 8:**
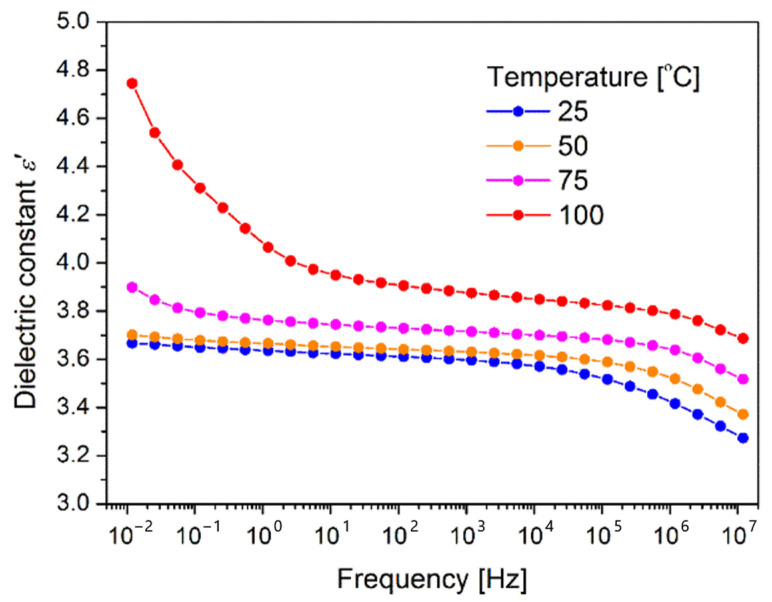
The dependence of the real part (ε′) of the dielectric permittivity on the applied voltage frequency for various temperatures for PI layer.

The measurements results of the dielectric constant dependence on the frequency of the applied AC voltage obtained for the different temperatures are shown in the Figure 8. The indoor electrification devices are in general working in the temperature range 25–100 °C, therefore temperature dependence was checked.

It can be seen, that dielectric constant decreases with a higher frequency what is easy to explain as PI is a polymer layer. In the case of polymers, the dielectric constant decreases gradually with the increasing frequency. Such a behavior is assigned to the frequency dependence of the polarization mechanisms. The dielectric constant is depended on the capability of the polarizable units in a polymer to orient in such a way to follow the oscillations of the applied alternating electric field. With the increased frequency, the orientational polarization decreases as the orientation of dipole moments require much longer time than electronic and ionic polarizations. Similar behavior was observed in the literature for the polyimide based samples [17].

The measurements at different temperatures indicate that with the increase of temperature the values of the dielectric constant increase, particularly at lower frequencies. At elevated temperatures, the charge carriers start to move at longer distances and a continuous connected network that allows the electric conduction is created. Therefore at higher temperature, the dielectric constant increases due to the high polarization which is a result of accumulation of the charge carriers at interfaces [36]. Such an effect is especially visible for the measurement presented in the Figure 8 for the sample measured at 100 °C.

Electrical devices operate usually at the frequency of 50 Hz and 60 Hz. One can see in Figure 8 that the value of dielectric constant in such frequency range and at room temperature is 3.6, close to that registered for the commercially available DuPont™ Kapton^®^ (Wilmington, NC, USA) polyimide films. The obtained result clearly shows that the developed method for application of polyimide binder is adequate for application as a dielectric layer.

### 3.7. Magnetic Measurements

**Figure 9:**
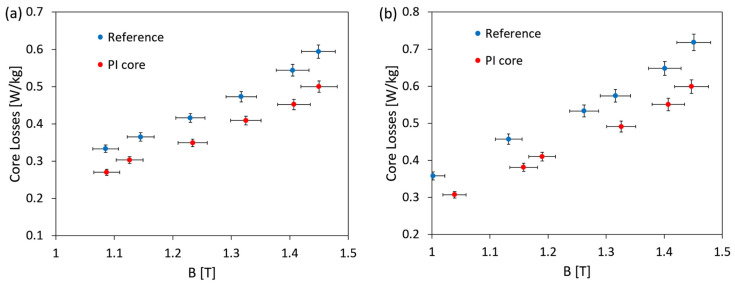
Core losses as a function of the operating flux density for the reference core and the toroidal magnetic core bonded with PI. Operating frequencies are: (**a**) 50 Hz and (**b**) 60 Hz.

Measurements of magnetic losses were performed for both 50 Hz and 60 Hz, operating grid frequencies, and the results are presented in Figure 9.

It is worth noting the relatively high core loss values obtained for wound model toroidal cores, which result from the specificity of the winding process and may be attributed to the formation of additional stresses when winding subsequent core layers with relatively small bending radii. Nevertheless, it can be observed that polyimide layer deposited between amorphous metal ribbons leads to lowering of the magnetic core losses in the whole operating flux density range. The average reduction of core losses is at the level of 0.07 W/kg (13%) and 0.09 W/kg (17%) for 50 Hz and 60 Hz, respectively. The applied PI layer hinders in an efficient way formation of the interlaminar eddy current, and as a result the core losses are decreased what confirms that the applied PI binder works well as an interlayer dielectric material.

### 3.8. Noise Analysis

**Figure 10:**
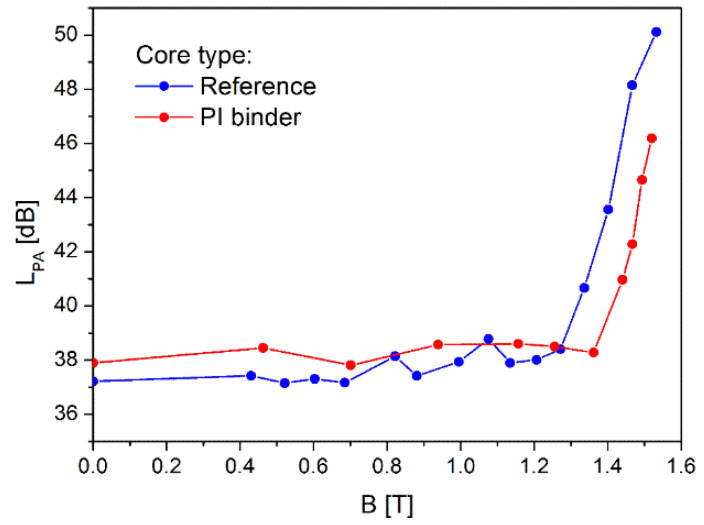
Sound pressure levels at operating frequency of 50 Hz as a function of the flux density measured for the reference and PI bonded cores.

Before the actual measurement, the background noise level was determined with the value of L_bgA_ = 38 dB(A). Next, the sound pressure levels (L_pA_) were measured during excitation of the cores from 0.4 T to the saturation flux density at 50 Hz, both for the core with PI based bonding and also for the reference core. The obtained results are presented in the Figure 10.

As shown in Figure 10, the core bonded with polyimide based binder exhibits the noise intensification above the background noise level for the much higher value of the operating flux density than for the measured reference core. In case of PI core sound pressure level starts to rise above background at flux of 1.36 T, whereas the reference core started to be noisy already at 1.25 T. The observed effect can be explained by the counteraction of the applied PI bonding layers against the magnetostriction, and as a result noise is clearly reduced. At the saturation flux of ca. 1.5 T, the difference in level of noise between the reference and PI bonded core is ca. 5 dB, therefore the reference core is twice as loud.

**Figure 11:**
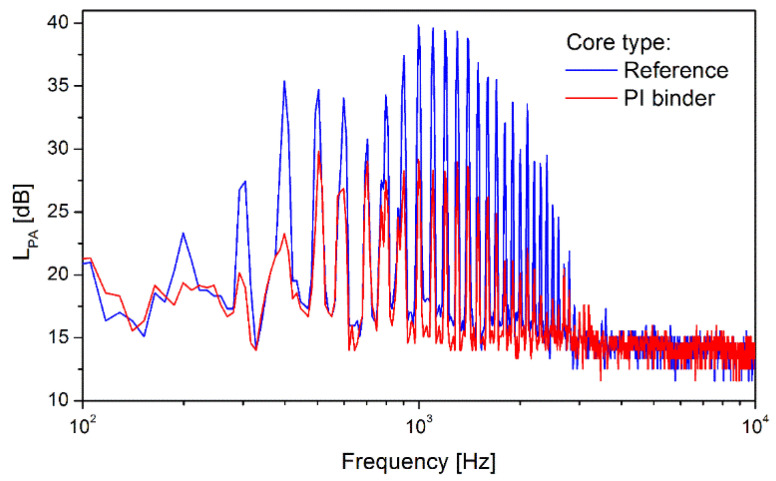
Frequency spectrum of sound pressure level for the reference and PI bonded cores. The cores were excited at the magnetic flux of 1.54 T and at the operating frequency of 50 Hz.

During the analysis also noise frequency spectrum in the range from 100 Hz to 10 kHz was identified and the result is presented in the Figure 11. The obtained results well proof the high effectiveness of the noise reduction by means of application of the polyimide binder. One can see that the PI bonded core produce lower level of sound pressure than the reference core, namely in the frequency bands at 400 Hz, 1 kHz and other harmonic frequencies (e.g., 1.1 kHz, 1.2 kHz, …). The observed behavior shows important application potential of the evaluated polyimide based binding material in order to reduce noise in the electrification devices based on the bonded amorphous steel tape, especially as new environmental regulations will require to reduce the noise of the electrical devices.

## 4. Conclusions

The main aim of the presented paper was to validate the applicability of a polyimide binder for consolidation of the amorphous metal tape used for fabrication of electrical devices. On the basis of the performed investigation the following conclusions can be drawn:

- PI binder resulted in high bonding strength (average value of 1520 kPa).
- Corrosion resistance was enhanced in relation to the raw amorphous metal.
- Applied deposition method resulted in formation of good dielectric layer (dielectric constant from 3.6–4.0 depending on temperature).
- Transformer core bonded with PI exhibited much lower core losses in relation to the reference (core losses of PI bonded core are 17% lower than losses of reference core).
- Core bonded with PI becomes noisy at noticeably higher flux density and is two times less noisy than the reference core.

Taking into account all the presented results, it can be concluded that the proposed method for preparation and deposition of polyimide layer, allowed to successfully bind together the amorphous metal ribbons. As a result, the mechanical integrity and strength of the produced magnetic core were improved, as well as main functional parameters, especially the core losses and audible noise were reduced, what is very positive from the application point of view.

## Figures and Tables

**Table 1:** Surface roughness of PI layers compared with reference samples.

Sample	R_a_
Reference_g	0.248 ± 0.065
Reference_m	1.046 ± 0.213
PI_dr_g	0.642 ± 0.116
PI_dr_m	1.559 ± 0.422
PI_an_g	0.392 ± 0.050
PI_an_m	1.571 ± 0.386

**Table 2:** Results of EDX examination of samples covered with PI and reference samples.

Sample	Element [At %]				
	Fe	Si	O	C	S
Reference_g	90	6	4		
Reference_m	92	4	4		
PI_dr_g	10	1	6	82	1
PI_dr_m	12	1	6	80	1
PI_an_g	11	1	5	83	
PI_an_m	12	1	5	82	

## Data Availability

The original contributions presented in the study are included in the article/Supplementary Material, further inquiries can be directed to the corresponding author/s.

## Supplementary Materials

The following supporting information can be downloaded at: https://www.mdpi.com/article/10.3390/polym16131840/s1, Figure S1: Glossy and matte sides of 2605 HB1M Metglas material; Figure S2: PI layer on: (a) glossy, and (b) matte side; Figure S3: Lines distribution on sample for surface roughness measurements; Figure S4: Visualization of the bonding of samples for mechanical examination; Figure S5: Sample prepared for the dielectric measurement: amorphous tape with applied layer of binder and round silver electrode on the top. Bottom copper disc is a part of the holder; Figure S6: Sample mounted in holder for dielectric test; Figure S7: Permeameter diagram (top), and core with secured primary and secondary windings and connected to testing device (bottom); Figure S8: Images of acoustic test assembly.
