# Supplementary material for: The Use of Polyimide as a Bonding Material to Improve the Mechanical Stability, Magnetic and Acoustic Properties of the Transformer Core Based on Amorphous Steel
Source: Polymers (Basel). 2024 Jun 28;16(13):1840. doi: 10.3390/polym16131840 (PMC11243792; doi:10.3390/polym16131840)
Supplement: Supplementary file 1 [file polymers-16-01840-s001.zip › polymers-3029159-supplementary.pdf]

Supplementary materials to article:

“The use of polyimide as a binding material to improve the mechanical stability, magnetic and acoustic properties of the transformer core based on amorphous steel”

by Jolanta Nieroda, et al.

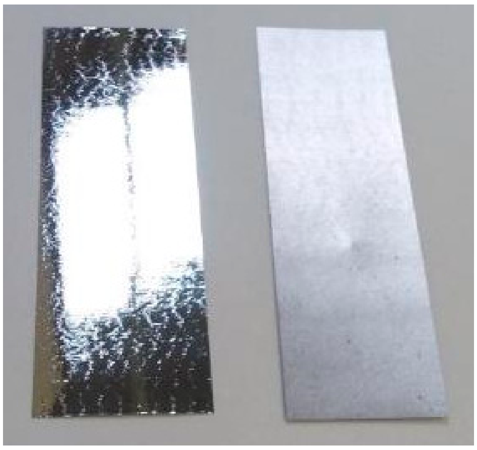

Figure S1. Glossy and matte sides of 2605 HB1M Metglas material.

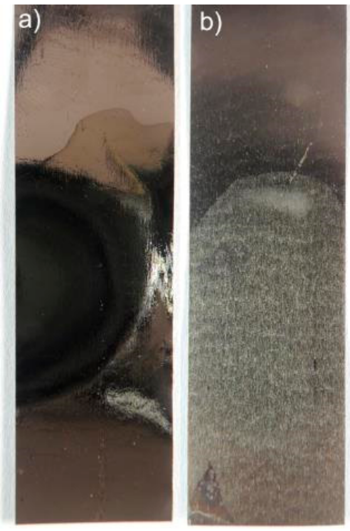

Figure S2. PI layer on: a) glossy, and b) matte side.

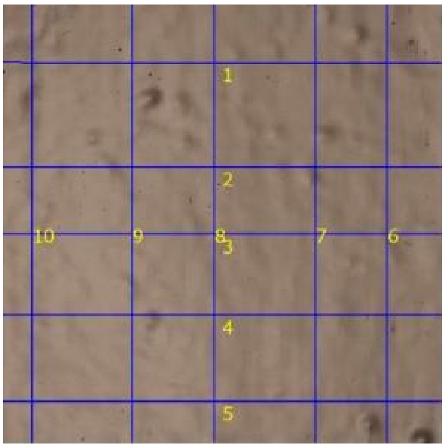

Figure S3. Lines distribution on sample for surface roughness measurements.

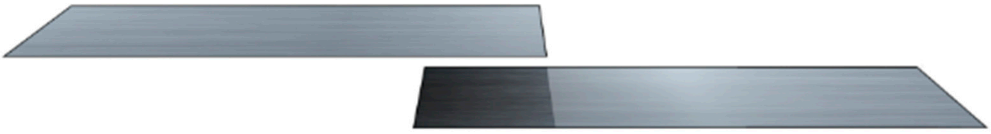

Figure S4. Visualization of the bonding of samples for mechanical examination.

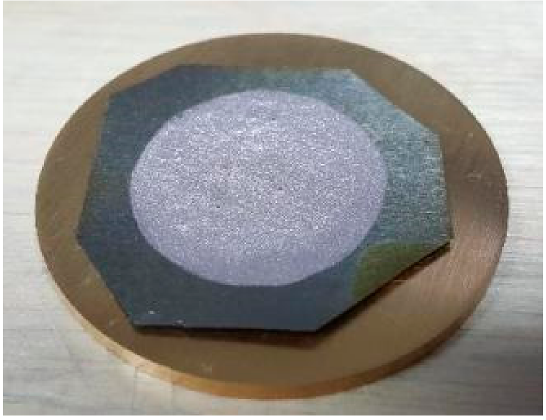

Figure S5. Sample prepared for the dielectric measurement: amorphous tape with applied layer of binder and round silver electrode on the top. Bottom copper disc is a part of the holder.

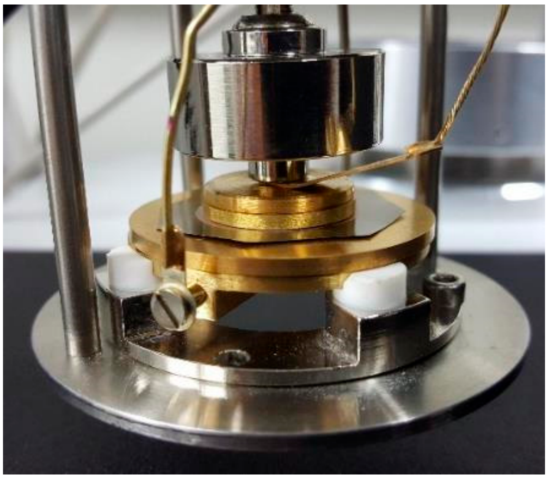

Figure S6. Sample mounted in holder for dielectric test.

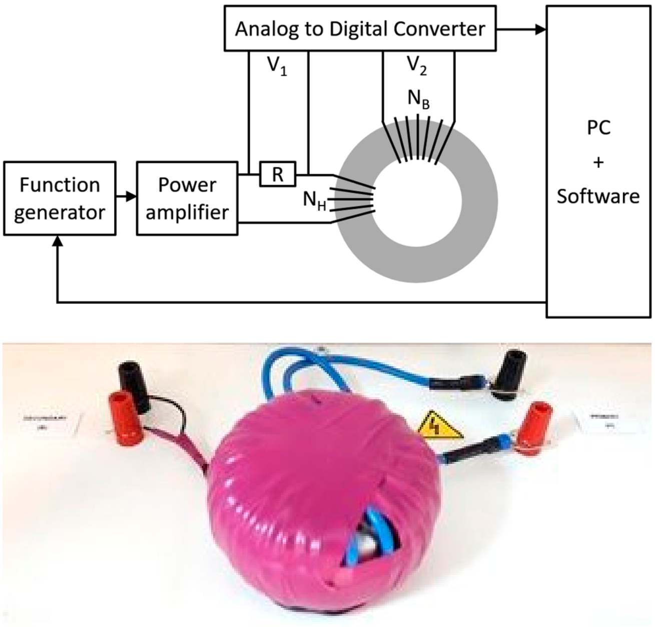

Figure S7. Permeameter diagram (top), and core with secured primary and secondary windings and connected to testing device (bottom).

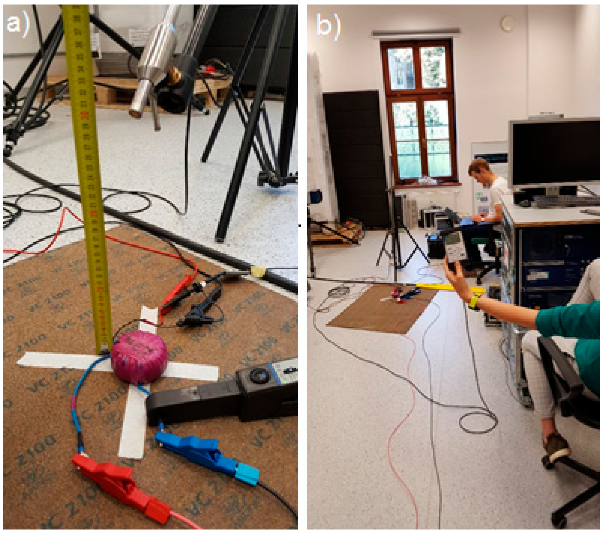

Figure S8. Images of acoustic test assembly.
